# Measurement agreement of the self-administered questionnaire of the Belgian Health Interview Survey: Paper-and-pencil versus web-based mode

**DOI:** 10.1371/journal.pone.0197434

**Published:** 2018-05-21

**Authors:** Elise Braekman, Finaba Berete, Rana Charafeddine, Stefaan Demarest, Sabine Drieskens, Lydia Gisle, Geert Molenberghs, Jean Tafforeau, Johan Van der Heyden, Guido Van Hal

**Affiliations:** 1 Department Epidemiology and public health, Sciensano, Brussels, Belgium; 2 Unit of Epidemiology and Social Medicine, University of Antwerp, Antwerp, Belgium; 3 Interuniversity Institute for Biostatistics and statistical Bioinformatics, University of Leuven, Leuven, Belgium; 4 Interuniversity Institute for Biostatistics and statistical Bioinformatics, University of Hasselt, Diepenbeek, Belgium; East Tennessee State University, UNITED STATES

## Abstract

Before organizing mixed-mode data collection for the self-administered questionnaire of the Belgian Health Interview Survey, measurement effects between the paper-and-pencil and the web-based questionnaire were evaluated. A two-period cross-over study was organized with a sample of 149 employees of two Belgian research institutes (age range 22–62 years, 72% female). Measurement agreement was assessed for a diverse range of health indicators related to general health, mental and psychosocial health, health behaviors and prevention with kappa coefficients and intraclass correlation (ICC). The quality of the data collected by both modes was evaluated by quantifying the missing, ‘don’t know’ and inconsistent values and data entry mistakes. Good to very good agreement was found for all categorical indicators with kappa coefficients superior to 0.60, except for two mental and psychosocial health indicators namely the presence of a sleeping disorder and of a depressive disorder (kappa≥0.50). For the continuous indicators high to acceptable agreement was observed with ICC superior to 0.70. Inconsistent answers and data-entry mistakes were only occurring in the paper-and-pencil mode. There were no less missing values in the web-based mode compared to the paper-and-pencil mode. The study supports the idea that web-based modes provide, in general, equal responses to paper-and-pencil modes. However, health indicators based upon factual and objective items tend to have higher measurement agreement than indicators requiring an assessment of personal subjective feelings. A web-based mode greatly facilitates the data-entry process and guides the completing of a questionnaire. However, item non-response was not positively affected.

## Introduction

Population surveys have traditionally used paper-and-pencil self-administered questionnaires to collect information on sensitive questions. However with the growth of internet use, web-based questionnaires have become an important alternative to paper-and-pencil questionnaires due to their many advantages [1;2]. For instance, the process of manual data-entry with its accompanying data-entry mistakes becomes unnecessary [3;4]. As well, web-based questionnaires can produce higher data quality since an automatic skipping and branching logic and warning messages in case of missing and implausible answers can be foreseen [3;4].

Web-based questionnaires cannot,however, be the sole mode of data collection for population surveys, as even in countries with high internet penetration, internet access and skills vary among demographic groups [5;6]. To overcome this limitation, mixed-mode data collection including a web-based and paper-and-pencil mode can be used. Mixing different modes in one survey, can lead to mode effects by simultaneously creating selection and measurement effects [7]. Selection effects can occur when respondents with different characteristics choose a different mode to complete the questionnaire. Measurement effects can occur if the mode influences how respondents understand the question, retrieve relevant information, make a judgment about the adequate response and finally choose the answer [8;9]. For instance, a web-based mode offers a greater opportunity to multitask since respondents are more likely to be engaged in several other activities while completing the questionnaire [10;11]. This might lead to “satisficing” behavior; respondents simply provide a satisfactory answer (e.g. answering don’t know or skipping the question) because an optimal response requires a substantial amount of cognitive effort [12;13]. As well, a web-based mode may limit the ability of the respondents to re-read the questions at their own pace, in their preferred order and to synchronize the answers [14;15]. Furthermore, a web-based mode can generate more honest responses since respondents can be transported into another virtual world wherein they forget their immediate surrounding [16]. In this way, it can create an illusion of privacy.

Mode effects have implications for the comparability of the data collected by different modes [8]. Recent meta-analyses and review studies of the comparability of electronic and paper-and-pencil modes generally found evidence for the equivalence across the modes [14;17;18]. However, other studies found differences in the reporting of general health [15], mental health [19;20] and sensitive health behaviors [21;22]. In a mixed-mode design, it is not possible to disentangle selection effects from measurement effects [7]. That is why, in the context of future mixed-mode data collection for the self-administered questionnaire of the Belgian Health Interview Survey (BHIS), a study with a repeated measures design was organized to test for measurement effects. More specifically, the aim of this study was to assess the measurement agreement between the newly developed web-based and the paper-and-pencil mode for several health indicators and to ascertain the extent to which the quality of the collected data varied between these modes.

## Methods

### Research design and study population

**Fig 1:**
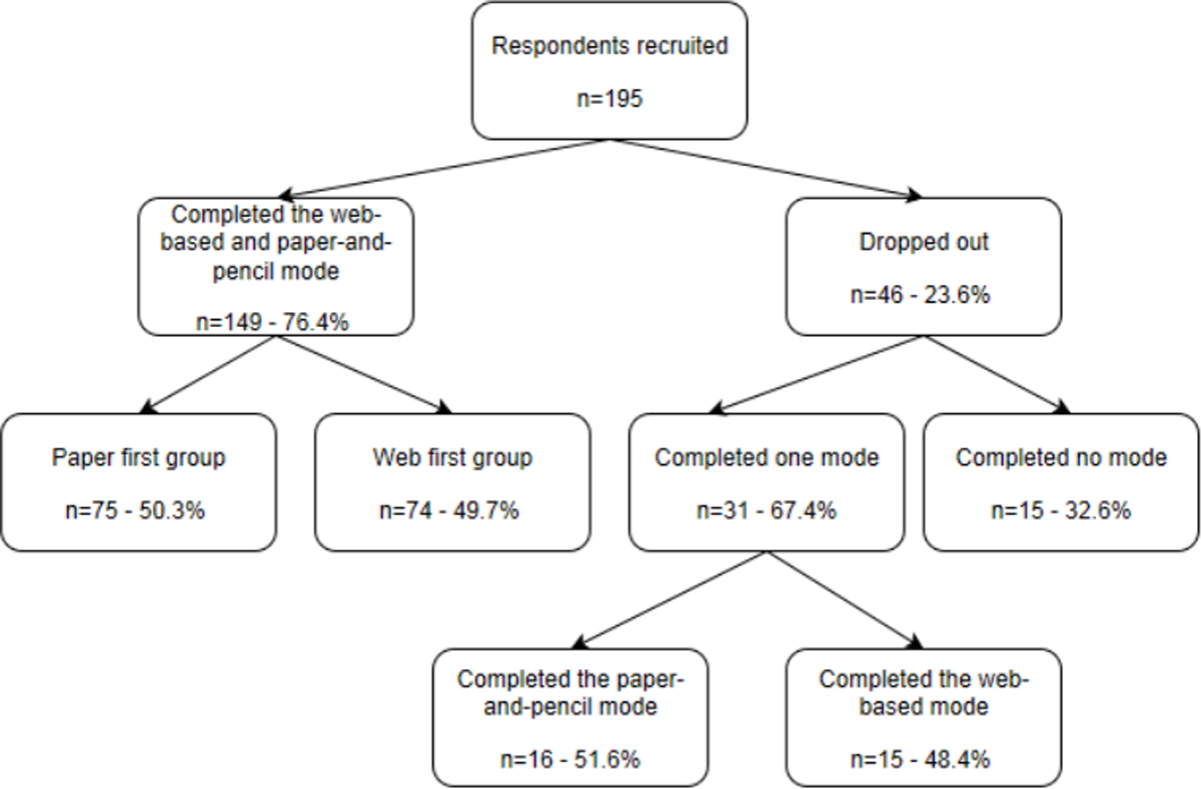
Overview of the sample size.

A two period cross-over design was used, in which respondents completed the questionnaire in both modes with a certain time interval in between. Respondents were recruited on a voluntary basis from a pool of 730 employees of two Belgian research institutes. The research protocol was submitted to the directors of the participating institutes for approval. No ethics committee was involved as this was an internal pilot study. The employees were informed about the objectives of the study in an e-mail before giving their written consent for participation. No benefits or risks were derived from participating in this study. The answers of the participants were kept anonymous as each participant had a unique ID code and the link between the name and the ID code was not accessible to the researchers. This link was deleted after the end of the data collection. In total 195 employees volunteered to participate. Half of the respondents were first assigned to the paper-and-pencil mode (paper first group) and the other half to the web-based mode (web first group). After two weeks the groups were switched: the paper first group received the web-based mode and inversely. Only respondents who completed the questionnaire by both modes were included in the final sample of 149 respondents. At end a response rate of 20.4% (149/730) was achieved. The median number of days between completing the questionnaire in the two modes was 14 days (minimum 2 and maximum 40 days) (**Fig 1**).

### Instrument

The questionnaire was based on the self-administered paper-and-pencil questionnaire of the BHIS 2013 [23] and could be completed in French or Dutch. The web-based questionnaire was developed to be as comparable as possible to the paper-and- pencil mode. Therefore, the questions were identical (similar wording and almost similar instructions) and the design was comparable (similar colors and lay-out). Still, the web-based mode was developed while applying the imbedded features of this mode such as automatic skipping and branching. Furthermore, soft warnings were given in case of missing values for the first question of every module and for filter questions and in case respondents gave inconsistent or implausible answers. As well, the web-based mode had a multipage design displaying only a few questions on every screen which differs from the paper-and-pencil questionnaire that allows a comprehensive view on the whole questionnaire. Web respondents were, however, able to go back in the questionnaire to change answers given to previous questions. After completing the last questionnaire respondents were asked if they had experienced a health change during the washout period.

The web-based questionnaire, developed using BlaiseIS 4.8 software, could be completed using a computer but not using a tablet or smartphone. Data from the web-based questionnaire were automatically saved in a database. Data collected with the paper-and-pencil questionnaire were entered manually using a program also developed with Blaise® software. A double data-entry was done in order to correct for data-entry mistakes. **Table 1**provides an overview of the indicators selected to assess the measurement agreement. These indicators are organized in 4 topics: general health, mental and psychosocial health, health behaviors, and prevention.

**Table 1:** Overview of the indicators.

Indicator	Type	# items/questions used	Instrument (*)
**General health**			
Self-rated health	Dichotomous	1	MEHM
Chronic health problem	Dichotomous	1	MEHM
Activity limitations	Dichotomous	1	MEHM
**Mental and psychosocial health**			
Mental distress	Dichotomous	12	GHQ-12
Eating disorder	Dichotomous	5	SCOFF
Depressive disorder	Dichotomous	13	SCL-90-R
Sleeping disorder	Dichotomous	3	SCL-90-R
Lifetime suicidal ideation	Dichotomous	1	
Quality of social support	Ordinal (3 categories)	3	OSLO Social support scale
Lifetime problematic alcohol consumption^a^	Dichotomous	4	CAGE
Vitality index	Continuous	4	SF-36 Vitality scale
**Health behavior**			
Alcohol drinking in the past 12 months	Ordinal (5 categories)	1	EHIS-2
Risky single occasion alcohol drinking	Ordinal (5 categories)	2	EHIS-2
Number of alcoholic drinks over the whole week^b^	Continuous	4	EHIS-2
Age at start drinking alcohol^c^	Continuous	2	
Smoking habits	Nominal (4 categories)	2	
Lifetime cannabis use	Dichotomous	1	EMCDDA
Sexual intercourse in the past 12 months^d^	Dichotomous	1	
Used contraception in the past 12 months^e^	Dichotomous	1	
**Prevention**			
Mammography in the past 2 years^f^	Dichotomous	2	
Cervix smear test in the past 3 years^f^	Dichotomous	2	
Ever being tested for HIV	Dichotomous	1	

(*) MEHM = Minimum European Health Module, GHQ-12 = 12-item General Health Questionnaire [24], SCL-90-R = Symptom Checklist-90-Revised [25], SF-36 = 36-item Short Form Health Survey, EHIS-2 = European Health Interview Survey wave 2, EMCDDA = European Monitoring Centre for Drugs and Drug Addictions

^a^ Lifetime problematic alcohol consumption is based on the CAGE questionnaire which contains the following questions: Have you ever felt you should cut down on your drinking? Have people annoyed you by criticizing your drinking? Have you ever felt guilty about your drinking? Have you ever had a drink first thing in the morning to really wake up or to get rid of a hangover? Since this indicator mainly reflects a self-image and provides no factual information about quantity, frequency, or pattern of drinking, it is included in the mental and psychosocial health section instead of the health behavior section. This indicator was not calculated for lifetime alcohol abstainers.

^b^ Number of alcoholic drinks over the whole week was only calculated for weekly drinkers.

^c^ Age at start drinking alcohol was only calculated for current and former alcohol drinkers.

^d^ Sexual intercourse in the past 12 months was only calculated for respondents who had at least on sexual intercourse in his/her lifetime.

^e^ Used contraception in the past 12 months was only calculated for women younger than 54 years old who had at least one sexual intercourse in the past 12 months.

^f^ Mammography in the past 2 years and cervix smear test in the past 3 years was only calculated for women.

### Statistical analyses

Statistical analyses were performed using the statistical package SAS® 9.3. The significance level for all the analyses was set at 5%, with corresponding 95% confidence intervals (CI).

#### Measurement agreement

For categorical indicators, kappa coefficients were estimated [26]. Simple kappa coefficients were calculated for binary and nominal indicators whereas linear weighted kappa coefficients were calculated for ordinal indicators. Weighted kappa coefficients take into account the greater disagreement between response categories that are further apart than for those that are closer together on an ordinal scale [27;28]. Linear weights were defined as w_i_ = 1-(i/(c-i)) where i is the difference between the response categories in the web-based mode and paper-and-pencil mode and c is the total number of categories of the indicator. For the interpretation, we followed the cutoffs proposed by Landis & Koch [26]: ≤0.00 = poor, 0.00–0.20 = slight, 0.21–0.40 = fair, 0.41–0.60 = moderate, 0.61–0.80 = good, 0.81–1.00 = very good agreement. In addition, percentages of exact (for binary, nominal and ordinal categorical indicators) and global agreement (only for ordinal categorical indicators) were calculated [29]. Exact agreement was estimated as the percentage of respondents who have the same category in both modes. Global agreement was calculated as the percentage of responses that fell within one category in the positive and negative direction. The percentages of agreement depend on the number of categories; they are expected to be higher for indicators with only a few categories.

Measurement agreement for continuous indicators was assessed using the intraclass correlation coefficient (ICC) [30]. The ICC measures the correlation between a single rating on a continuous measure using the web-based mode and a continuous measure using the paper-and-pencil mode [4]. A score above 0.80 is usually sought in mode comparison, with 0.70 considered as an acceptable value [31]. ICC is based on mean-centered versions of the indicators and is insensitive to respondent’s tendency to provide consistently higher responses in one mode compared to the other [4;31]. For this reason, Wilcoxon signed ranked tests were calculated to detect the presence of differences between both modes.

Kappa and ICC coefficients were calculated overall and by order group (web first or paper first group). In this paper the overall kappa and ICC coefficients are presented. However in case of a difference between the order groups, the coefficients by order group are mentioned. Further, the kappa and ICC coefficients were calculated with and without respondents who said they experienced a health change (n = 11) but since it had almost no effect, it was decided to use the sample including all respondents.

#### Data quality

The quality of the data was assessed by evaluating the missing, ‘don’t know’ and inconsistent values. The latter was defined as an answer that should not have been given according to the skipping and branching logic or as an answer that was inconsistent with other answers. ‘Don’t know’ is a non-substantive answer since it can be seen as a way of refusing to answer a question [32]. The quantification of the values was done by counting the total number of these values separately for both modes of data collection. Furthermore, the mean number of missing, ‘don’t know’ and inconsistent values by questionnaires were calculated for both modes and the differences between the modes were evaluated by performing a Wilcoxon signed rank test.

Additionally, paper-and-pencil surveys require manual data-entry and this may generate mistakes and hence, have a negative impact on the data quality. For this reason, a double data-entry was performed. In case inconsistencies were found, they were resolved by checking the paper-and-pencil questionnaire. The number of data-entry mistakes was assessed by counting the total number of data-entry mistakes per data encoder.

## Results

### Characteristics of the respondents

About 72% of the respondents were female and 57% were younger than 40 years. The age range was 22 to 62 years. No gender or age differences between the order groups were detected (**Table 2**).

**Table 2:** Characteristics of the respondents (n = 149).

	All (n = 149)		Web first (n = 74)		Paper first (n = 75)		
	n	%	n	%	n	%	*p*^*a*^
**Sex**							0.96
Male	42	28.2	21	28.4	21	28.0	
Female	107	71.8	53	71.6	54	72.0	
**Age**							0.46
< 40	85	57.0	40	54.1	45	60.0	
≥ 40	64	43.0	34	45.9	30	40.0	

^*a*^ *p* value for difference between web first and paper first group from chi square test.

### Measurement agreement

#### General health

For two indicators a very good agreement was found, with a kappa coefficient of 0.92 (95% CI: 0.85–1.00) for chronic health problems and of 0.84 (95% CI: 0.69–0.99) for activity limitations (**Table 3**). For self-rated health there was somewhat lower but still good agreement (kappa = 0.74 (95% CI: 0.53–0.96)). About 97% of the respondents had the same response category in both modes for these indicators. The kappa coefficients calculated within each order group showed lower agreement in the web first group compared to the paper first group for self-rated health and for activity limitations. However, there was at least moderate agreement between both modes (kappa≥ 0.55).

**Table 3:** Kappa coefficients and percentages of agreement between paper-and-pencil and web-based mode for categorical indicators.

	Paper-and-pencil		Web-based		Kappa (95% CI)	Exact agreement (%)^a^	Global agreement (%)^b^
	n	%	n	%			
**General health**							
**Self-rated health (n = 148)**					0.74 (0.53–0.96)	96.6	
Good to very good health	138	93.2	137	92.6			
Fair, bad to very bad health	10	6.8	11	7.4			
**Chronic health problem (n = 146)**					0.92 (0.85–1.00)	97.3	
Yes	33	22.6	35	24.0			
No	113	77.4	111	76.0			
**Activity limitations (n = 148)**					0.84 (0.69–0.99)	97.3	
Yes	15	10.1	13	8.8			
No	133	89.9	135	91.2			
**Mental and psychosocial health**							
**Mental distress (n = 148)**					0.61 (0.48–0.74)	81.1	
Yes	61	41.2	65	43.9			
No	87	58.8	83	56.1			
**Eating disorder (n = 148)**					0.78 (0.61–0.95)	95.9	
Yes	15	10.1	15	10.1			
No	133	89.9	133	89.9			
**Depressive disorder (n = 146)**					0.52 (0.32–0.71)	87.7	
Yes	22	15.1	22	15.1			
No	124	84.9	124	84.9			
**Sleeping disorder (n = 146)**					0.50 (0.35–0.64)	77.4	
Yes	53	36.3	46	31.5			
No	93	63.7	100	68.5			
**Lifetime suicidal ideation (n = 148)**					0.86 (0.76–0.95)	94.6	
Yes	38	25.7	38	25.7			
No	110	74.3	110	74.3			
**Perceived quality of social support (n = 149)**^**c**^					0.66 (0.55–0.76)	77.9	100.0
Low	20	13.4	24	16.1			
Moderate	90	60.4	83	55.7			
High	39	26.2	42	28.2			
**Lifetime problematic alcohol consumption (n = 137)**					0.69 (0.52–0.85)	91.2	
Yes	24	17.5	22	16.1			
No	113	82.5	115	83.9			
**Health behavior**							
**Alcohol drinking in the past 12 months (n = 149)**^**c**^					0.86 (0.81–0.92)	83.9	100.0
No drinking in past 12 months	14	9.4	14	9.4			
Less than once a month	14	9.4	14	9.4			
Monthly	38	25.5	41	27.5			
Weekly	68	45.6	64	43.0			
Daily	15	10.1	16	10.7			
**Risky single occasion alcohol drinking (n = 147)**^**c**^					0.84 (0.76–0.91)	86.4	98.6
Abstainer/infrequent drinker	14	9.5	14	9.5			
Never or not in the past 12 months	81	55.1	76	51.7			
Less than monthly	31	21.1	33	22.4			
Monthly	17	11.6	19	12.9			
Weekly	4	2.7	5	3.4			
**Smoking habits (n = 149)**					0.96 (0.91–1.00)	98.0	
Daily	8	5.4	9	6.0			
Occasional	10	6.7	7	4.7			
Former	25	16.8	26	17.4			
Never	106	71.1	107	71.8			
**Lifetime cannabis use (n = 149)**					1.00 (1.00–1.00)	100.0	
Yes	44	29.5	44	29.5			
No	105	70.5	105	70.5			
**Sexual intercourse in the past 12 months (n = 140)**					0.93 (0.83–1.00)	98.6	
Yes	124	88.6	124	88.6			
No	16	11.4	16	11.4			
**Used contraception in the past 12 months (n = 79)**					0.95 (0.84–1.00)	98.7	
Yes	68	86.1	69	87.3			
No	11	13.9	10	12.7			
**Prevention**							
**Mammography in the past 2 years (n = 107)**					0.95 (0.88–1.00)	98.1	
Yes	27	25.2	27	25.2			
No	80	74.8	80	74.8			
**Cervix smear test in the past 3 years (n = 104)**					0.80 (0.65–0.95)	94.2	
Yes	84	80.8	88	84.6			
No	20	19.2	16	15.4			
**Ever being tested for HIV (n = 146)**					0.93 (0.87–0.99)	96.6	
Yes	86	58.9	83	56.8			
No	60	41.1	63	43.2			

For each indicator, statistics were calculated among respondents who gave an answer in both modes.

^a^ Percentage of respondents who have the same response category in the web-based and paper-and-pencil mode.

^b^ Percentage of respondents who have the same or within one response category in the positive or negative direction in the web-based and paper-and-pencil mode.

^c^ Weighted kappa coefficients were calculated instead of simple kappa coefficients for ordinal categorical indicators. In addition to percentages of exact agreement, we also calculated percentage of global agreement for these indicators.

#### Mental and psychosocial health

For lifetime suicidal ideation a very good agreement was found (kappa = 0.86 (95% CI: 0.76–0.95)) (**Table 3**). Four other indicators showed good agreement with kappa coefficients varying between 0.61 (95% CI: 0.48–0.74) for mental distress and 0.78 (95% CI: 0.61–0.95) for the presence of an eating disorder. The presence of a depressive disorder (kappa = 0.52 (95% CI: 0.32–0.71)) and of a sleeping disorder (kappa = 0.50 (95% CI: 0.35–0.64)) exhibited only moderate agreement. 77.4% to 95.9% of the respondents had the same response category in both modes. For the ordinal categorical indicator quality of social support, all respondents reported the same response category or stayed within one response category in the positive or negative direction. The kappa coefficients calculated in each order group showed somewhat lower agreement for the presence of an eating disorder in the web first group compared to the paper first group and for the presence of a sleeping disorder and lifetime problematic alcohol consumption in the paper first group compared to the web first group. However the agreement was still at least moderate between both modes (kappa ≥ 0.57) except for the presence of a sleeping disorder (kappa = 0.36 (95% CI: 0.14–0.58)).

The continuous indicator vitality index had an ICC value of 0.79 (95% CI: 0.72–0.84) which indicates that the agreement was acceptable (**Table 4**). No significant difference between the two modes was observed. The ICC coefficients were similar when doing the analyses in each order group.

**Table 4:** Intraclass correlation between the paper-and-pencil and web-based mode for continuous indicators.

	Paper-and-pencil	Web-based		
	Mean (SD)	Mean (SD)	ICC (95% CI)	*p*^*a*^
**Mental and psychosocial health**				
**Vitality index (n = 148)**	56.38 (16.42)	56.69 (18.70)	0.79 (0.72–0.84)	0.60
**Health behavior**				
**Number of alcoholic drinks over the whole week (n = 75)**	7.85 (7.00)	7.59 (6.21)	0.89 (0.83–0.93)	0.91
**Age at start drinking alcohol (n = 131)**	17.53 (3.48)	17.32 (3.43)	0.91 (0.88–0.94)	0.14

For each indicator, statistics were calculated among respondents who gave an answer in both modes.

^a^ *p* value derived from Wilcoxon signed rank test.

#### Health behaviors

For all six categorical health behavior indicators very good agreement was found (**Table 3).** The kappa coefficients ranged between 1.00 (95% CI: 1.00–1.00) for lifetime cannabis use and 0.84 (95% CI: 0.76–0.91) for risky single occasion alcohol drinking. The percentages of exact agreement indicate that 83.9% to 100% of the respondents had the same response category in the web-based mode as in the paper-and-pencil mode. Concerning the two ordinal indicators alcohol drinking in the past 12 months and risky single occasion alcohol drinking, 100% and 98.6% of the respondents, respectively, gave the same response category or remained within one response category in the web-based and paper-and-pencil mode. When considering kappa coefficients calculated in each order group, equal results were obtained.

The ICC coefficients for the continuous indicators showed high agreement for the number of alcoholic drinks over the whole week (0.89 (95% CI: 0.83–0.93)) and for the age at starting drinking alcohol (0.91 (95% CI: 0.88–0.94)) **(Table 4).** No significant differences between the two modes were identified. The ICC coefficients were similar when we did the analyses for every order group.

#### Prevention

For mammography in the past 2 years and ever being tested for HIV a very good agreement was found with kappa coefficients of, respectively, 0.95 (95% CI: 0.88–1.00) and 0.93 (95% CI: 0.87–0.99) (**Table 3).** For cervix smear test in the past 3 years somewhat lower but still good agreement was found (kappa = 0.80 (95% CI: 0.65–0.95)). 94.2% to 98.1% of the respondents had the same response category in both modes for the prevention indicators. The kappa coefficients indicated lower agreement for cervix smear test in the past 3 years in the web first group compared to the paper first group. However, the level of agreement was still good (kappa = 0.65 (95% CI: 0.37–0.93)).

### Data quality

Although the total number of missing values was low in both modes, it was higher in the web-based mode (228 (1.3%)) compared to the paper-and-pencil mode (104 (0.6%)) (**Table 5**). No significant differences were found in the mean number of missing values between the questionnaires in both modes. The total number of ‘don’t know’ values was somewhat higher in the paper-and-pencil mode (93 (3.2%)) compared to the web-based mode (82 (2.8%)) but no significant differences in the mean numbers were found. In the paper-and-pencil mode, there were 12 (1.3%) inconsistent values, while no such values were detected in the web-based mode because of the integrated controls and automatic skipping and branching logic. The two data encoders made 132 data-entry mistakes in total. Data encoder 1 made more mistakes (117 (0.7%)) than data encoder 2 (15 (0.1%)).

**Table 5:** Comparison of missing values, ‘don’t know’ values and inconsistent values between the paper-and-pencil and web-based mode and number of data entry mistakes in the paper-and-pencil mode (n = 149).

	Paper-and-pencil		Web-based		
	Total count (%)	Mean (SD)	Total count (%)	Mean (SD)	*p*^*a*^
Missing values	104 (0.6)^b^	0.70 (2.72)	228 (1.3)^b^	1.53 (7.75)	0.49
‘Don't know‘ values	93 (3.2)^c^	0.62 (1.45)	82 (2.8)^c^	0.55 (1.09)	0.67
Inconsistent values	12 (1.3)^d^	0.08 (0.27)	0	0	0.0005
Data-entry mistakes					
Data encoder 1	117 (0.7)^e^				
Data encoder 2	15 (0.1)^e^				

^a^ *p* value derived from Wilcoxon signed rank test.

^b^ Percentage of missing values out of the total number of questions that needed to be replied. Character items not included.

^c^ Percentage of ‘don’t know’ values out of the total number of questions for which ‘don’t know’ was a possible answer category.

^d^ Percentage of inconsistent values out of the total number of questions for which an inconsistent answer could be given. Inconsistent values were not possible in the web-based mode.

^e^ Percentage of data entry mistakes out of the total number of data entries. Character items not included. The double data entry was done for 146 of 149 completed paper-and-pencil questionnaires due to the late return of 3 paper-and-pencil questionnaires.

## Discussion

This study showed generally a strong agreement between the web-based and the paper-and-pencil mode. For general health indicators good to very good agreement was observed. This is consistent with the findings of Hoebel et al. [8] who found no differences in the prevalence rates for general health indicators between a web-based and paper-and-pencil health interview survey and of Ritter et al. [33] who found that respondents answered similarly in these modes for self-report general health instruments. All behavior indicators showed very good agreement. This is in agreement with the results of Vergnaud et al. [4] who found high measurement agreement for variables related to tobacco use. Hoebel et al. [8] also found no differences in the prevalence rates for tobacco use and alcohol consumption between these modes. For the three prevention indicators good to very good agreement was found. This is again in line with Hoebel et al. [8] who found no differences between the web-based and paper-and-pencil mode for participation in influenza vaccination which can be seen as a prevention indicator.

For mental and psychosocial health good to very good agreement was found for six indicators and moderate agreement was observed for two indicators namely the presence of a sleeping disorder and of a depressive disorder. This is in line with a systematic review study that found generally high reliability between electronic and paper-and-pencil modes for psychiatric self-report instruments [17]. The moderate agreement found for depressive and sleeping disorder could be related to the recall period of only one week of the SCL-90-R instrument [34]. Since the washout period in this study was two weeks, it is possible that respondents experienced mood swings or sleeping variation between completing both questionnaires. The variation between health topics in measurement agreement could be due to the nature of the questions as all indicators for which very good agreement was found are based upon factual and objective items whereas the indicators for which moderate agreement was found require assessing personal subjective feelings.

As expected, the web-based mode offered advantages regarding data quality. In the paper-and-pencil mode, respondents gave some answers that should not have been given according to the branching logic and answers that were inconsistent with other answers. Such problems were not reported in the web-based mode due to integrated controls and automatic branching and skipping logic. Furthermore, the process of manual data-entry and the accompanying mistakes were avoided. However, there were no less missing values in the web-based mode. On the contrary, slightly more missing values were generated but this was not a statistically significant difference. Other studies generally found less missing values in a web-based mode compared to a paper-and-pencil mode [3;4;18]. This difference might be explained by the fact that our respondents were allowed to skip questions. Studies that also didn’t enforce answers as well found slightly more missing values in the web-based mode [35;36].

This study has some limitations. A convenience sample of the employees of two research institutes was used. These people are generally in good health, part of the working-age population, mainly highly educated and probably familiar with completing questionnaires in both modes. Consequently, it should be acknowledged that this sample excluded people who do not routinely access the internet. Due to these factors, the sample may not be representative for the general population. Nevertheless, web-based questionnaires in mixed-mode surveys are more likely to attract younger and highly educated people with internet access [37]. This study tested measurement agreement for BHIS indicators which are aggregated indicators based upon multiple questions/items of existing health instruments and that combine multiple response categories of questions. This might have masked potential differences between modes. A two-week washout period prevented that answers given the first time would be recalled and influenced the answers given the second time [38]. However, since this study was organized during the holiday period some variability in the wash-out period occurred (2–40 days). Nevertheless other studies that tested measurement agreement reported comparable variability in washout periods [3;4;39] and a study that compared test-retest reliability of health status instruments using a two-day or two-week washout period found no time interval effect [40]. Furthermore, respondents could indicate if they experienced a health change during the washout period since this could have affected the agreement [3].

In conclusion, this study supports the idea that web-based modes provide, in general, equal responses as paper-and-pencil modes. A web-based mode greatly facilitates the data-entry process and guides the completing of a questionnaire, however, item non-response was not positively affected. Even with the limitation of having a sample with a majority of highly educated and internet familiar people, the agreement between the two modes was quite substantial to conclude that mixed-mode data collection including a paper-and-pencil and web-based questionnaire could be undertaking without impacting the comparability of the estimates.

## Supporting information

S1 Dataset (XLSX)
